# Supplementary material for: Complexome profiling of the Chlamydomonas psb28 mutant reveals TEF5 as an early PSII assembly factor
Source: Plant Cell. 2025 Mar 17;37(6):koaf055. doi: 10.1093/plcell/koaf055 (PMC12164590; doi:10.1093/plcell/koaf055)
Supplement: koaf055_Supplementary_Data [file koaf055_supplementary_data.zip › psb28_interactive.zip!/plots/intensity/qualityPlots/peptides/CPN10_Cre03.g178450.t1.2.html]

### Cre03.g178450.t1.2

Trivial name: CPN10  
SeqCoverage [median]: 74.70  
Mass: 10.77 kDa  
  
 

| Sequence | MissedCleavage | Mass [Da] | wtIntensity | muIntensity | Score |
| --- | --- | --- | --- | --- | --- |
| EGDKVLLPEYGGSQIK | 1 | 1731.90 | 5583.59 | 22092.81 | 81.31 |
| LIPLLDR | 0 | 838.53 | 0.00 | 8499.63 | 73.04 |
| NKDGDLLPTNVK | 1 | 1312.70 | 0.00 | 7487.67 | 77.67 |
| NKDGDLLPTNVKEGDK | 2 | 1741.88 | 850.07 | 0.00 | 49.34 |
| NKDGDLLPTNVKEGDKVLLPEYGGSQIK | 3 | 3026.59 | 0.00 | 1867.30 | 40.45 |
| TAGGVLLPESVTQK | 0 | 1398.77 | 10667.34 | 47525.51 | 186.46 |
| VLIDKVQAVSK | 1 | 1198.73 | 11673.18 | 22672.69 | 97.91 |
| VLLPEYGGSQIK | 0 | 1302.72 | 2838.17 | 32483.21 | 112.83 |
| VNEGIVVAVGPGR | 0 | 1265.71 | 2090.83 | 19315.61 | 188.28 |
| VNEGIVVAVGPGRR | 1 | 1421.81 | 8392.98 | 20937.25 | 102.66 |
|  |  |  | 42096.2 | 182881.7 |  |
